# Lay beliefs about the badness, likelihood, and importance of human extinction

**DOI:** 10.1038/s41598-026-39070-w

**Published:** 2026-02-20

**Authors:** Matthew Coleman, Lucius Caviola, Joshua Lewis, Geoffrey P. Goodwin

**Affiliations:** 1 Department of Psychology, Harvard University, Cambridge, MA USA; 2 Department of Psychology, Northeastern University, Boston, MA USA; 3 Institute for Technology and Humanity, University of Cambridge, Cambridge, UK; 4 Department of Marketing, New York University, New York, NY USA; 5 Department of Psychology, University of Pennsylvania, Philadelphia, PA USA

**Keywords:** Human extinction, Catastrophic risk, Likelihood estimates, Global priorities, Cross-cultural, Environmental social sciences, Philosophy, Social sciences

## Abstract

Human extinction would mean the end of humanity’s achievements, culture, and future potential. According to some ethical views, this would be a terrible outcome for humanity. But what are the public’s beliefs about human extinction? And how much do people prioritize preventing extinction over other societal issues? Across five empirical studies (*N* = 2,147; U.S. and China), we find that people consider extinction prevention a societal priority and deserving of greatly increased societal resources. However, despite estimating the likelihood of human extinction to be 5% this century, people believe that the chances would need to be around 30% for it to be the very highest priority (U.S. medians). In line with this, people consider extinction prevention to be only one among several important societal issues. We also find that people’s judgments about the relative importance of extinction prevention appear relatively fixed and hard to change by reason-based interventions.

**Supplementary Information:**

The online version contains supplementary material available at 10.1038/s41598-026-39070-w.

## Introduction

Human extinction would be the most consequential event in human history. It would mean the end of humanity’s achievements, culture, and future potential. In this article, we explore what the general public believes about human extinction and why society does not prioritize mitigating extinction risks more.

Studying beliefs about human extinction can provide important insights for both policy and public education. If public views diverge from the assessments of experts in areas such as nuclear security, biorisk, artificial intelligence, climate change, and human extinction more generally, this may point to public misconceptions that need to be addressed. At the same time, such divergences may also highlight areas where expert communities should engage more effectively with public concerns. Such gaps are practically relevant because they can mean that the public may not understand or accept policy proposals recommended or enacted by experts, potentially sowing avoidable conflict and undermining policy uptake. In either case, understanding these differences can inform how extinction risks are communicated and how society should decide the level of priority such risks warrant. Public beliefs can also signal whether society should do significantly more or less to mitigate such risks.

Studying human extinction beliefs is also psychologically interesting because human extinction would be a unique type of event^1^. Usually, people can rely on personal experience, emotions, or imagination to guide their risk judgments^2–7^. Human extinction, in contrast, is unprecedented, abstract, vast, and extremely uncertain^8^. Beliefs about human extinction might tell us something about what it means to be a responsible civilization—one that recognizes the risks it faces and takes steps to reduce them. Setting aside longstanding religious traditions of apocalyptic thought—many of which describe catastrophic but temporary upheavals rather than the permanent end of humankind—the secular notion of human extinction is surprisingly recent. Only in the Enlightenment, with the development of fields such as geoscience, demography, and probabilism, thinkers started recognizing and assessing the possibility that the human species could end and whether that would be desirable or not^9–13^.

Humanity could go extinct in many different ways: through natural or human-caused means, gradually or abruptly, painfully or painlessly. We are interested in what people believe about human extinction in general, not about their views on specific types of extinction events. Still, we also explore which causes of extinction they have in mind when considering human extinction. According to several experts, the most likely causes of human extinction this century are risks caused by advanced technology^14–16^. These include risks caused via global nuclear war, runaway climate change, engineered pandemics, and uncontrolled artificial intelligence. However, experts have diverse beliefs about the likelihood of human extinction^17^. A recent survey of 80 extinction risk experts found a median estimate of a 6% chance of human extinction this century^14^.

Philosophical theories disagree on how bad human extinction would be^18,19^. The answer may depend on how extinction would occur. Some ethical theories do not consider human extinction bad per se; instead, they consider it bad insofar as it causes widespread suffering^20,21^. Other ethical theories consider extinction one of the worst possible outcomes not only because of the potential for immediate suffering but also because of the loss of all of humanity’s collective future potential^15,22,23^.

One intellectual approach to assessing the importance of reducing the chances of human extinction is to quantify both the value of the future^22,24^ and the risk of losing it^15^. The rationale is that since human extinction would cause an unprecedented, enormous loss in value (depending on one’s moral views), even a relatively small likelihood of its occurring would justify prioritizing its prevention as long as society could meaningfully do something about it. Most theories of rationality consider the likelihood of an event in addition to its value to be important inputs to decision-making. The most prominent is expected value theory^25,26^. To calculate an action’s expected value, one should multiply the likelihood of each possible outcome of that action by its value and sum the resulting products together. According to the theory, one should then choose actions with the highest expected value. That means, for example, that it could be considered more rational to save 1000 lives with a 10% chance than to save one life with a 100% chance. Under certain assumptions, the application of expected value theory to extinction prevention can lead to a surprising conclusion—the view that, given the large stakes of extinction, even a relatively small reduction in risk likelihood could make extinction prevention the most important societal issue (cf.^27^). In this paper, we use expected-value reasoning as a normative benchmark, not as a descriptive theory of lay decision-making. We note, however, that there are alternative normative decision frameworks, especially for the extinction context (e.g., the lexical precautionary principle^28^).

Here, we investigate whether laypeople’s opinions approximately align with expected value theory. We do so by surveying people’s empirical and normative beliefs about extinction and attempting to change their beliefs to observe potential changes in their judgments about the importance of reducing extinction risks. We hypothesize that people are reluctant to strictly prioritize human extinction prevention above other societal issues. If true, this would not mean that people consider human extinction unimportant or not even *a* priority, just that they do not consider it *the highest societal priority*.

There are multiple ways by which people might form their prioritization judgments. One possibility is that these judgments are driven largely by empirical beliefs about the likelihood of human extinction, humanity’s ability to prevent it, and the value of doing so. In this case, people may approximate expected value reasoning or other theories of rational choice, including those that emphasize risk aversion^26,29–33^, by integrating beliefs about both the probability and the disvalue of extinction. Alternatively, people’s judgments about the relative priority of human extinction may be relatively fixed, and thereby less sensitive to their beliefs about the likelihood and disvalue of human extinction.

We investigated our research questions in five studies through two empirical approaches across two cultures. First, we measured people’s empirical (i.e., factual) and normative beliefs about human extinction in three surveys: Study 1a (*N* = 185; U.S. via Prolific), Study 1b (*N* = 143; China via Lucid), and Study 2 (*N* = 251; U.S. via Prolific). Second, we experimentally manipulated the apparent severity of human extinction (Study 3, *N* = 1,000; U.S. via Prolific) and participants’ familiarity with expected value reasoning (Study 4, *N* = 568; U.S. via Prolific) to see whether these changes would elicit a change in participants’ prioritization judgments. Note that we present the study results by topic, which sometimes deviates from the order in which the studies were conducted.

## Results

In our initial studies, we assessed people’s beliefs about the potential risks and disvalue associated with human extinction.

### Human extinction is mostly considered bad

Do most people even believe human extinction would be bad? After all, if human extinction were not bad, then it would not be important to prevent it. A previous study found that 78% of U.S. Americans believe human extinction would be bad^34^. We find converging evidence in our studies. In particular, we found that the majority of participants (Study 1a U.S.: 68.6%, Study 1b China: 69.9%, Study 2 U.S.: 66.1%) reported that if humans went extinct in the next 100 years it would be a bad outcome. Meanwhile, very few reported that it would be good (Study 1a U.S.: 6.5%, Study 1b China: 9.1%, Study 2 U.S.: 6.8%) and the remainder reported that it would be neither good nor bad (Study 1a U.S.: 24.9%, Study 1b China: 21.0%, Study 2 U.S.: 27.1%). We found similar results using a more granular measure in which we asked participants how bad they would find it if humanity went extinct relatively soon using a 7-point scale from *1 (not bad at all)* to *7 (extremely bad)* (Study 2). The average response was significantly above the scale midpoint (*M* = 5.56, *SD* = 1.71; *t*(250) = 14.46, *p* < .001, *d* = 0.92, 95% CI = [5.35, 5.77]). 73.7% of participants responded above the midpoint. These results demonstrate that most people view human extinction as a bad outcome, although it is noteworthy that people differ in *how* bad they believe it would be.

In the Supplementary Materials, we report additional findings about the assumptions participants had in mind when answering our questions about extinction. The most strongly endorsed reasons for why extinction would be bad were that it would cause the loss of all human progress, it would fail to uphold our duty to protect future generations, and that it would cause pain to the people dying from the extinction event. Participants were roughly equally split in assuming that the extinction process would be sudden or gradual. However, most participants assumed extinction would occur through a catastrophic event that involved a great deal of suffering and killed all living humans. Participants considered extinction worse if it were to occur painfully than painlessly. However, on average, they tended to consider it bad regardless of whether suffering was involved.

### Reducing the risks of human extinction is considered possible

Do people believe it is possible to reduce the risks of human extinction? If there were nothing society could do to meaningfully reduce human extinction risks, it would make sense not to prioritize it more. To date and to our knowledge, no work has directly examined the perceived tractability of reducing human extinction risks. To answer this question, we asked participants if they believe there are actions humanity could take that would at least slightly reduce human extinction risks (Study 1a). We found that 92.4% responded “yes”, with only 7.6% responding “no”. We also asked participants (Study 4) on a 7-point scale from *1 (strongly disagree)* to *7 (strongly agree)* whether they believe it is possible for society to meaningfully reduce human extinction risk this century. The average response was significantly above the scale midpoint (*M* = 4.92, *SD* = 1.60; *t*(567) = 13.69, *d* = 0.57, 95% CI = [4.79, 5.05]). 66.9% of participants responded above the midpoint. Finally, we asked participants whether a variety of actors and institutions could, if they wanted to, take actions to meaningfully reduce the risks of human extinction on a 7-point scale from *1 (not at all)* to *7 (to a large extent)* (Study 2). They rated governments the highest (*M* = 5.78, *SD* = 1.51), followed by for-profit companies (*M* = 4.87, *SD* = 1.91), social movements (*M* = 3.99, *SD* = 1.69), non-profit organizations (*M* = 3.90, *SD* = 1.56), influential individuals (*M* = 3.89, *SD* = 1.75), and typical individual citizens (*M* = 3.04, *SD* = 1.75). Taken together, these findings suggest that people generally do believe there are ways to meaningfully reduce human extinction risks.

### Human extinction is considered a societal priority but not above all others

Next, we asked people directly whether they believe human extinction prevention should be a societal priority. First, we asked them whether they agreed or disagreed with the statement that “reducing the risks of human extinction should be the very highest priority of governments and society in general” (Study 1a). 62.7% of respondents agreed. We also found that people believed a much greater share of global income (median: 11%, *M* = 23.1%, *SD* = 24.7) should be spent on reducing human extinction risks than the share they estimate is currently spent (median: 2%, *M* = 7.28%, *SD* = 12.4). These results show that people do, at least in the most general terms, consider reducing extinction risks very important. However, this question asked about human extinction in isolation. Thus, it is unclear how much more important they consider it relative to other societal issues when faced with direct trade-off questions.

To address this question, we explicitly assessed how human extinction should be prioritized relative to other societal issues (Study 2). U.S. participants ranked different societal issues from *1 (most important)* to *8 (least important)*. Participants ranked reducing risks of human extinction (*M* = 5.32, *SD* = 2.73) as less important than improving healthcare (*M* = 2.63, *SD* = 1.55), reducing poverty (*M* = 2.84, *SD* = 1.64), improving education (*M* = 3.45, *SD* = 1.66), reducing homelessness (*M* = 4.18, *SD* = 1.77), and improving law and order (*M* = 5.05, *SD* = 2.18), but more important than improving transportation (*M* = 5.86, *SD* = 1.41) and reducing risks of car crashes (*M* = 6.67, *SD* = 1.39). This suggests that people consider the prevention of human extinction as *a* societal priority, yet do not see it as the very highest priority. Next, we explored why this might be.

### Human extinction is considered relatively likely

How likely do people think human extinction this century is? If it were the case that people believed human extinction is extremely unlikely (e.g., below 1 in 10,000 this century), that could explain why they do not prioritize it over other societal issues, i.e., they simply do not find it pressing enough. Given that extinction this century would affect every person in every country, we surveyed both U.S. (Studies 1a and 2) and Chinese (Study 1b) participants to estimate the perceived chances of human extinction this century^1^. Both the U.S. and China are global superpowers with an outsized impact in determining the likelihood of extinction risks coming to fruition. Moreover, the two countries tend to contrast in key cultural values such as individualism and collectivism^35^. Because, arguably, collectivistic societies may be less worried about technological risks^36^ and tend to be more optimistic about the quality of the future^37^, we thought that Chinese participants might have lower estimates of the likelihood of human extinction.

The median U.S. participant estimated the chances of human extinction this century at 5% (Study 1a: *M* = 20.1%, *SD* = 26.7; Study 2: *M* = 15.4%, *SD* = 20.6). Contrary to our expectation, the median Chinese estimate was slightly higher at 15% (*M* = 22.5%, *SD* = 22.8). However, based on a Wilcoxon rank-sum test, the U.S. and Chinese estimates did not significantly differ from each other (*W* = 11846, *p* = 0.10). A majority of participants estimated a chance of 1% or greater (Study 1a: 73.5%; Study 2: 76.7%; 75.5% Chinese). Future research could explore how robust these lay estimates are. It is worth noting that people’s estimates could heavily depend on the precise wording of the question^38^ and that many people struggle with probabilistic reasoning. In an earlier study, only 3.1% of participants reported a time frame of 100 years or less for human extinction^39^.

### Human extinction is not considered likely enough to be the very highest priority

Most people consider the chances of human extinction this century to be 1% or higher, but at the same time, they do not view extinction prevention as the very highest societal priority (see Table S1 in Supplementary Materials). Would they consider human extinction the most important societal priority if it were more likely? And if so, how likely^2^ would it need to be?

**Fig. 1:**
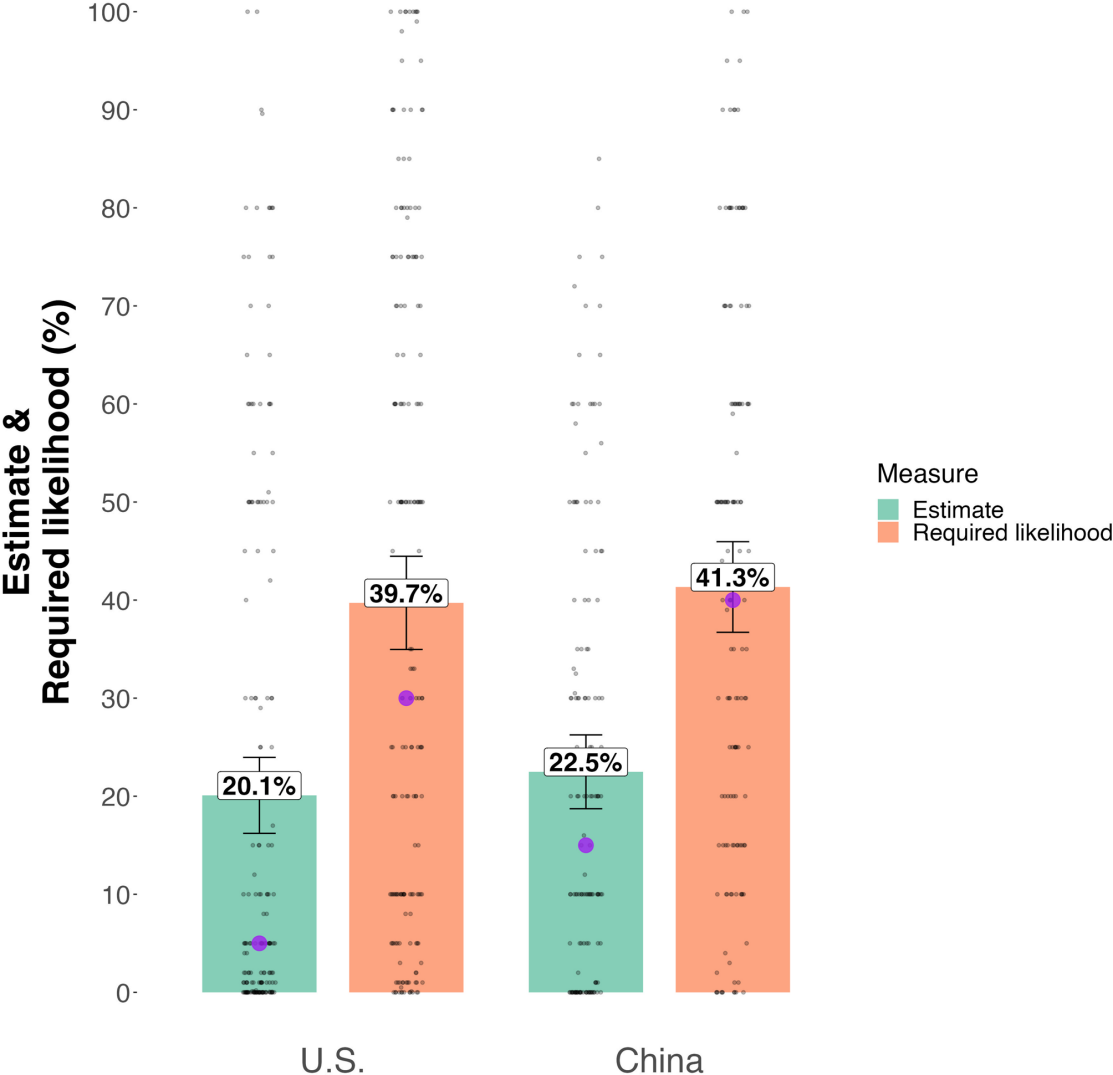
Reported likelihood estimates of human extinction this century and ‘required likelihoods’ for human extinction to be the top societal priority. Results for U.S. participants (Study 1a) and Chinese participants (Study 1b). Most participants reported higher required likelihoods than likelihood estimates, suggesting they think human extinction is not likely enough to be the top societal priority. Numbers in boxes indicate mean values, purple dots indicate medians, and error bars indicate 95% confidence intervals.

To test this, we asked participants for their beliefs about the *required likelihood* of human extinction for it to be the top societal priority. Specifically, they were asked how likely human extinction would need to be this century in order to be *the* very highest priority of governments and society in general. We found U.S. participants (Study 1a) believed that human extinction would need to be far more likely than their median estimate of 5% to be the top societal priority. The median required likelihood was 30% (*M* = 39.7%, *SD* = 32.8). Moreover, 71.4% of U.S. participants’ required likelihoods were greater than their likelihood estimates, implying they did not find extinction likely enough to be the highest priority. Similarly, the median required likelihood for Chinese participants was 40% (*M* = 41.3%, *SD* = 28.0), which based on a Wilcoxon rank-sum test, did not differ from the U.S. sample (*W* = 12522, *p* = 0.41; see Fig. 1). Moreover, 64.3% of Chinese participants had required likelihoods that were greater than their likelihood estimates. Thus, despite relatively high likelihood estimates, most participants in both countries did not see extinction prevention as the very highest priority.

Overall, despite reporting relatively high likelihood estimates for human extinction, most people in the U.S. and China seem to consider extinction not likely enough to be the very highest priority.

### Reducing required likelihoods does not affect prioritization judgments

Are people’s judgments about the relative priority of preventing human extinction based on their beliefs about the likelihood of human extinction and the likelihood required for human extinction to be the top priority? In an experiment, we attempted to reduce people’s required likelihood to observe whether this increased their prioritization of extinction risk.

In our pre-registered experiment (Study 3), all U.S. participants were asked how likely extinction would have to be this century for it to be the very highest priority. Our intervention was a modified version of the unit-asking technique, usually used to address scope insensitivity in the charitable giving domain^40–42^. In the experimental condition, participants were first asked how likely the risk of everyone dying in a city of one million people would have to be for reducing that risk to be the very highest priority of governments and society in general. We expected that this task would subsequently make participants lower their required likelihood for human extinction by allowing them to recognize that the stakes of human extinction are much higher. In other words, directly contrasting a smaller (yet still severe) risk with an even larger risk could make the severity of the human extinction risk even more apparent. The participants in the control condition did not answer the prior question about the risk of one million people dying. Next, participants in both conditions completed a prioritization measure that asked how they would prefer to allocate limited resources between reducing risks to human extinction and five current societal issues (healthcare, education, law and order, transportation, homelessness), using a scale from − 3 *(strongly prioritize that current societal issue)* to + 3 *(strongly prioritize reducing risks that could cause human extinction)*.

We found that the intervention successfully reduced people’s required likelihoods for human extinction to be the very highest priority from a median of 20% (*M*_*control*_ = 29.2%, *SD*_*control*_= 28.3) to 9.5% (*M*_*interv*_ = 22.8%, *SD*_*interv*_ = 30.6; *W* = 153963, *p* < 0.001). Further, the intervention reduced the proportion of participants with required likelihoods greater than their estimates from 75.1% in the control condition to 62.7% in the experimental condition (*χ2* = 17.52, *p* < 0.001). This shows that the intervention increased participants’ endorsement of the view that human extinction is sufficiently likely, according to their own standards, to be the top societal priority. Crucially, however, despite successfully decreasing participants’ required likelihoods, the intervention did not affect how much people prioritized human extinction relative to other societal issues (aggregated score of the five societal issues) when making direct comparisons (*M*_*control*_ = 0.54, *SD* = 1.44; *M*_*interv*_ = 0.64, *SD* = 1.49; *t*(992) = − 1.14, *p* = 0.25, *d* = 0.07).

These results suggest that people do not primarily base their relative prioritization judgments on their stated beliefs about the likelihood of human extinction and the required likelihood that has to be met for it to be the very highest priority. If they were doing this, then by successfully lowering people’s required likelihoods, we would expect them, at least in part, to also change their explicit prioritization beliefs.

### Teaching expected value reasoning does not affect prioritization judgments

In Study 4, we tested whether people prioritize human extinction reduction more after receiving training in expected value reasoning applied to societal risk management. One possibility is that people simply do not know about expected value reasoning and would apply it if they learned about it. The aim of this study, therefore, was to test whether people who have received training would lower their required likelihood for human extinction to be the top priority and increase the relative priority they place on human extinction.

In the 10–15 min training program, participants learned how to make estimates about an uncertain event’s likelihood, the magnitude of its consequences if the event occurred, and how to multiply the two to calculate the expected value (see Supplementary Materials). In particular, participants learned that, according to expected value reasoning, even unlikely risks should sometimes be prioritized—as long as the stakes are sufficiently large. For example, participants were asked to imagine an unlikely risk of a volcano eruption that could cause thousands of people to die unless there was sufficient safety preparation. In this particular example, the potential harm was high enough to justify the costs of mitigating the damage despite the low probability of an eruption. The training program included comprehension checks and three practice trials with feedback to confirm that participants understood how to apply expected value reasoning. The clear majority of participants (85.4%) responded correctly to at least two of the three practice trials (the control condition did not receive these practice trials). After the training, participants were told to respond to the remaining study questions by explicitly applying expected value reasoning. Similar to the previous studies, the questions asked about the likelihood, the required likelihood, and the relative societal importance of human extinction.

The expected value training intervention did not affect participants’ required likelihoods (control median = 22%, intervention median = 20%; *W* = 41650, *p* = 0.46) nor their relative prioritization of human extinction compared to current societal issues (*M*_*control*_ = − 0.68, *SD*_*control*_ = 1.42; *M*_*interv*_ = − 0.69, *SD*_*interv*_ = 1.39; *t*(562) = 0.06, *p* = 0.952). However, the intervention did have an impact on some of the beliefs people held about human extinction. In particular, we found that expected value training significantly *decreased* people’s likelihood estimates of human extinction this century from a median of 12%^3^ (*M*_*control*_ = 24.1%, *SD*_*control*_ = 26.3) to 5% (*M*_*interv*_ = 15.2%, *SD*_*interv*_ = 21.4; *W* = 47582, *p* < 0.001). As a consequence and contrary to our hypothesis, the intervention significantly *increased* the proportion of participants with required likelihoods greater than their estimated likelihoods from 60.9% in the control condition to 77.0% (χ2 = 16.23, *p* < 0.001).

These findings demonstrate that teaching people expected value reasoning may not be sufficient to increase the relative priority they place on human extinction reduction. Why is this? One possibility is that participants did not know how to transfer their newly gained knowledge about expected value reasoning to the context of human extinction reduction. Another is that people by default (e.g., even in the control condition) already approximated expected value reasoning, such that teaching them expected value reasoning did not affect their judgments. Yet another possibility is that people’s judgments about the relative prioritization of human extinction are relatively fixed and cannot easily be affected by asking people to reason from their other relevant beliefs by applying expected value calculations.

## General discussion

Our studies demonstrate that most people find human extinction bad and believe there are meaningful ways for society to reduce its likelihood. Accordingly, they consider the prevention of human extinction a societal priority. They believe governments should invest many more resources in extinction prevention than they currently do. However, while most people view human extinction prevention as *a* societal priority, they do not consider it *the very highest priority* that should come before everything else. Instead, they view human extinction as one among several other important societal priorities, such as healthcare, poverty, and education. And while we found similar patterns of responses in the U.S. and China, we also found noteworthy individual differences in people’s beliefs about human extinction.

We also found that a clear majority of people believe human extinction is at least 1% likely this century. The median estimate was 5% among U.S. participants and 15% among Chinese participants. These are remarkably high estimates that are also comparable to the estimates of extinction experts^14,15^. However, most people do not consider human extinction likely enough for it to be a higher priority than other societal issues. When asked for the required likelihood for it to be the very highest priority, the median answer was approximately 30% (30% in Study 1a, 40% in Study 1b, 20% in Study 3’s control condition, 22% in Study 4’s control condition). These are again remarkably high numbers and it is unclear why people believe human extinction must be this likely for it to be the very highest priority. By contrast, many experts regard extinction prevention as warranting top-priority status even at far lower likelihood thresholds, given the enormous stakes involved.

How do people form their judgments about the relative prioritization of human extinction? One possibility is that they base their judgments on their (stated) beliefs about the likelihood, required likelihood, tractability, and disvalue of human extinction. Our studies did not find much evidence that people form their prioritization judgments in this way. First, when we induced a decrease in people’s required likelihood for extinction to be the top priority, this did not change their prioritization judgments. (Still, the intervention did reduce the share of participants whose required likelihood exceeded their estimate, from 75.1% to 62.7%, yet even then, prioritization judgments remained unchanged.) This provides some evidence that people do not reach their prioritization judgments by contrasting their belief about how likely extinction is with their belief about how likely it would need to be for it to be the very highest priority. Second, when we taught people expected value reasoning, this did not change their prioritization judgments either. One plausible explanation is that people’s prioritization judgments are relatively fixed. Thus, these judgments cannot be changed much by asking people to rely explicitly on their beliefs about the likelihood and disvalue of extinction (i.e., by applying expected value reasoning). Despite these considerations, our studies cannot rule out that people do, at least in part, rely on their beliefs when forming their prioritization judgments about human extinction. In general, more research is needed to understand how people form these judgments and what assumptions and intuitions they rely on. Moreover, while we focused here on expected value theory as a normative baseline, future research could examine how public judgments align with alternative frameworks, such as risk-sensitive models (e.g., allowing for greater risk aversion or rank-dependent weighting) and precautionary principles (e.g., the lexical precautionary principle;^28^).

One drawback of our research is the possibility that we might not have adequately measured, or perhaps missed entirely, certain beliefs that influence people’s judgments about extinction. One notable limitation is that we did not rigorously examine people’s moral preferences toward currently existing people and how such beliefs could partly explain why they perceive ongoing societal issues as equally or even more pressing than reducing human extinction. For example, people might consider it a much higher priority to alleviate current suffering than to safeguard the future of humanity (see Study 1a in the Supplementary Materials;^43–45^. In addition, there are likely other non-consequentialist values driving people’s judgments.

Another limitation concerns framing: we asked about extinction in general terms while providing canonical examples, but future work should directly compare generic versus concrete (single-cause) framings to test how much scenario specificity alters judgments. It would also be valuable to compare extinction prevention with other catastrophic (non-extinction) risks to assess its relative priority within a broader risk-portfolio context.

While we asked participants whether they believed it is possible to reduce human extinction, we did not probe these beliefs in a fine-grained way. For instance, some people may believe that although risk reduction is possible, the potential impact is so marginal that investing heavily in it is not worthwhile. Others may think humanity could in principle reduce extinction risk but that identifying effective actions is extraordinarily difficult, given the diffuse, multi-causal, and uncertain nature of such risks. In other words, people can regard risk as tractable in theory while doubting our ability to target effective interventions in practice. Still others may believe that human extinction is inevitable, such that even temporary reductions in risk will not meaningfully alter the long-term outlook. Finally, it is possible that people’s stated beliefs do not entirely reflect their true beliefs and that hidden beliefs drive their prioritization judgments. Future research could explore in more detail what drives people’s prioritization judgments and test different strategies to raise awareness about human extinction risks and other pressing societal issues.

### Methods

All studies were approved by institutional review boards at Harvard University (CR14-3025-11) or New York University (#IRB-FY2020-4536), and all participants gave their informed consent. All studies were performed in accordance with relevant guidelines and regulations.

For Study 1b conducted in China, the consent form and survey materials were professionally translated into Mandarin and reviewed for clarity and cultural appropriateness by a native speaker. Participants were recruited via Lucid, an established online sampling platform with experience recruiting respondents in China. The study was considered minimal risk by the approving IRB in part because participation was anonymous and the questionnaire did not collect identifying or sensitive personal information (e.g., name, contact information, or political affiliation).

### Study 1a/b

As part of a larger study, we asked participants in both the U.S. (conducted in July 2022) and China (conducted in February 2023) whether human extinction would be good, bad, or neither. We also asked how likely they believe human extinction is in the next 100 years (U.S.) or this century (China), from 0% to 100% and how likely the chances of human extinction would need to be this century in order for it to be the very highest priority of governments and society in general, also from 0% to 100%.

We included several additional measures in the U.S. sample only. In one measure, we provided a list of reasons why human extinction may be good or bad and asked participants to endorse each possible reason from *1 (the very weakest reason)* to *7 (the very strongest reason)*. Additionally, we asked them to rank specific extinction risks from most to least likely. We also asked participants if there are actions humanity could take to at least slightly reduce the risks of human extinction, with binary “Yes” or “No” response options. We also asked participants whether they agree or disagree with the statement that “reducing the risks of human extinction should be the very highest priority of governments and society in general”. We also asked both the percentage of global income they estimate is currently spent and the percentage that *should* be spent on reducing human extinction risks, each from 0% to 100%.

U.S. participants were recruited through Prolific Academic. A total of 198 participants were recruited. Thirteen participants were excluded for failing the attention check, leaving a final sample of 185 participants (87 female, *M*_*age*_ = 39.26, *SD*_*age*_ = 15.65).

Chinese participants were recruited through Lucid. A total of 202 participants were recruited. Fifty nine participants were excluded for failing the attention check, leaving a final sample of 143 participants (54 female, *M*_*age*_ = 34.64, *SD*_*age*_ = 9.09).

### Study 2

Participants for this pre-registered study (https://aspredicted.org/3L9_3YL*)*, conducted in March 2024, were recruited through Prolific Academic. A total of 258 U.S. participants were recruited. Seven participants were excluded for failing to complete the study, leaving a final sample of 251 participants (122 female, *M*_*age*_ = 35.62 years, *SD*_*age*_ = 12.02).

To measure whether and how bad participants find human extinction, they were asked “Would it be good or bad if humanity went extinct (such that no human being will ever live anymore) in the next 100 years?” and responded with either “good”, “neither good nor bad”, or “bad”. Next, participants were asked “How bad would it be if humanity went extinct (such that no human being will ever live anymore) in the next 100 years?” and responded on a 7-point Likert scale from 1 (Not bad at all) to 7 (Extremely bad). Afterward, participants were asked “How likely do you personally think it is that humanity will go extinct (such that no human being will ever live anymore) in the next 100 years?” and responded with a percentage between 0 and 100. Participants were then asked to rank a list of eight societal issues in terms of their importance for society to prioritize. The issues were presented in a randomized order and included reducing risks of human extinction in the next 100 years, reducing risks of car crashes, improving healthcare, reducing poverty, improving education, improving law and order, improving transportation, and reducing homelessness. Participants were instructed to rank the issues from most important (top) to least important (bottom).

To measure the perceived tractability of reducing human extinction risks, participants were asked “To what extent do you think the following types of actors could, if they wanted, take actions to meaningfully reduce the risks of human extinction?” and responded on a scale from 1 (Not at all) to 7 (To a large extent) for each of the following types of actors: governments, for-profit companies, non-profit organizations, social movements, influential individuals, and individual typical citizens.

We also included several additional measures about participants’ assumptions when responding to the previous questions, including the extent to which they were thinking about various possible causes of human extinction, and the badness of human extinction depending on whether it happened immediately vs. gradually and painfully vs. painlessly. These measures and their results are included in the Supplementary Materials.

### Study 3

Participants for this pre-registered study (https://aspredicted.org/L4P_9K8*)*, conducted in January 2023, were recruited through Prolific Academic. A total of 1000 U.S. participants were recruited (508 female, *M*_*age*_ = 38.64, *SD*_*age*_ = 14.23).

The study used a 2-cell between-subjects design. The main dependent variable was people’s required likelihoods for human extinction this century in order for it to be the top societal priority.

Participants in the experimental condition were first presented with a scenario involving a risk that, if it happened, would certainly cause the entire population of a city with one million people to die at some point this century. Participants were then asked to provide a percentage between 0% and 100% that represents the minimum chances that would make reducing its likelihood the very highest priority of governments and society in general.

Then, all participants were presented with a scenario involving a risk that, if it happened, would certainly cause the entire world’s population to die (i.e., human extinction) this century. Participants were then asked to provide a percentage between 0% and 100% that represents the minimum chances that would make reducing its likelihood the very highest priority of governments and society in general.

Afterwards, participants were presented with different societal issues and asked to prioritize allocating limited resources between reducing risks that could cause human extinction (the entire human population being killed, e.g., from nuclear war, an asteroid impact, advanced artificial intelligence, an engineered pandemic, etc.) and addressing other societal issues. The issues presented were healthcare, education, law and order, transportation, and homelessness. Participants responded on a 7-point Likert scale ranging from − 3 *(strongly prioritize that current societal issue)* to + 3 *(strongly prioritize reducing risks that could cause human extinction)*.

Next, participants were asked to provide their personal estimate of the likelihood that humanity will go extinct this century (e.g., from nuclear war, an asteroid impact, advanced artificial intelligence, an engineered pandemic, etc.) from 0% to 100%. Finally, participants completed an attention check.

### Study 4

Participants for this study, conducted in March 2023, were recruited through Prolific Academic. A total of 605 U.S. participants were recruited. Thirty seven participants were excluded for failing the attention check and/or comprehension check, or for not completing the entirety of the survey, leaving a final sample of 568 participants (257 female, *M*_*age*_ = 39.77, *SD*_*age*_ = 14.05).

A 2-cell between-subjects design was used for this study. Participants were randomly assigned either to an intervention condition in which they completed an expected value training or to a control condition in which they did not complete this training.

First, participants in the intervention condition completed an approximately 10–15 min training on expected value by going through examples on estimating the chances of an event and its impact, multiplying them together, and using that answer to guide their decisions (see Supplementary Materials). The control condition did not complete this training.

Next, participants were asked to estimate the likelihood of humanity going extinct (e.g., from nuclear war, an asteroid impact, advanced artificial intelligence, an engineered pandemic, etc.) this century from 0% to 100%. Additionally, they were asked to indicate the minimum chances of human extinction this century in order for them to believe that reducing its likelihood should be the very highest priority for governments and society, also from 0% to 100%.

Participants were also asked to prioritize allocating resources between reducing risks that could cause human extinction (e.g., from nuclear war, an asteroid impact, advanced artificial intelligence, an engineered pandemic, etc.) and addressing other societal issues, such as healthcare, education, law and order, transportation, and homelessness, using a scale from *− 3 (strongly prioritize the societal issue)* to *+ 3 (strongly prioritize reducing risks that could cause human extinction)*.

Finally, participants were asked if they agree that it is possible for society to meaningfully reduce the risk of human extinction this century, on a 7-point scale from *1 (strongly disagree)* to *7 (strongly agree)*.

## Supplementary Information

Below is the link to the electronic supplementary material.

Supplementary Material 1

## Data Availability

Reports of all measures, manipulations, and exclusions, and all data, analysis code, and experimental materials for all studies are available for download in the Open Science Framework repository at: https://osf.io/kg9fw.

## Footnotes

1 In Studies 1a and 2, we asked “in the next 100 years”, whereas in Study 1b, we asked “this century”.

2 It is not obvious how the likelihood of human extinction should influence the importance of reducing it. On the one hand, the more risk there is, the more valuable it is to reduce that risk to 0%, all else equal. Moreover, it might be easier to achieve a given amount of risk reduction from higher amounts (e.g., 91% to 90%) than from lower amounts (1% to 0%). Perhaps people share this assumption. On the other hand, there are also reasons to assume that a high likelihood of human extinction would make it less valuable to reduce the risk. In particular, perhaps a high likelihood of human extinction this century indicates a higher likelihood of extinction in the ensuing centuries (i.e., if all else is *not* equal). If so, reducing this century’s risk alone would be less valuable in expectation because humanity would likely go extinct anyway in a subsequent century (Thorstad, 2022). We nonetheless assume that participants believe a greater likelihood of extinction increases the importance of reducing that risk.

3 The median human extinction likelihood estimate of 12% in the control condition was higher than in previous studies. It is possible that this is because the study was run several months later, meaning current events may have affected these estimates. Another possible explanation is that in the other studies the item assessing participants’ perceived likelihoods appeared after other extinction-related measures and that answering those other measures affected their likelihood estimates.
