# Supplementary material for: Lay beliefs about the badness, likelihood, and importance of human extinction
Source: Sci Rep. 2026 Feb 20;16:10020. doi: 10.1038/s41598-026-39070-w (PMC13021939; doi:10.1038/s41598-026-39070-w)
Supplement: Supplementary file 1 — Supplementary Material 1 [file 41598_2026_39070_MOESM1_ESM.pdf]

SUPPLEMENTARY MATERIALS

Study 1a: additional results

Badness, tractability, and likelihood

Badness

Would it be good or bad if humanity went extinct (no human being will ever live anymore) in the next 100 years?

[ ] Good
[ ] Neither good nor bad
[ ] Bad

Tractability

Do you think there are actions we as humanity could take, if we wanted, that would at least slightly reduce the risks of human extinction?

[ ] Yes
[ ] No

Likelihood

How likely do you personally think it is that humanity will go extinct (no human being will ever live anymore) in the next 100 years?

Please write a percent between 0 - 100. Decimals are allowed.

___%

Table S1. Proportion of participants who believe that human extinction this century would be a bad outcome (i.e., "bad"), that there are actions humanity could take to at least slightly reduce the risks of human extinction (i.e., "tractable"), that the likelihood of human extinction this century is at least 1%.

Variable(s)	Proportion of participants (%)
Bad	68.7%
Tractable	92.4%
Likelihood >= 1%	73.5%
Bad + tractable	63.2%
Bad + likelihood >= 1%	47.6%
Tractable + likelihood >1%	69.2%
Bad + tractable + likelihood >= 1%	45.4%

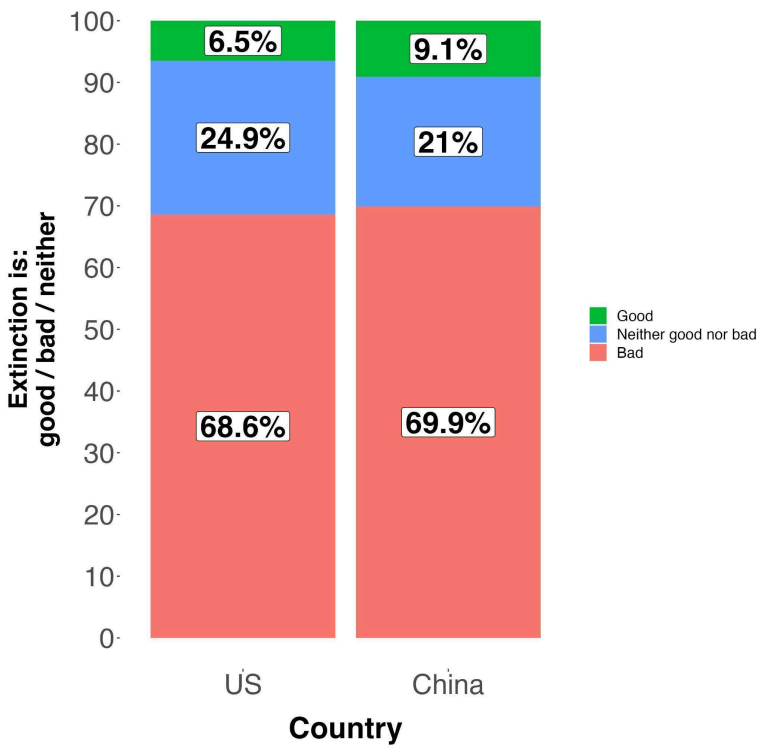

Figure S1. Proportion of participants in the U.S. (Study 1a) and China (Study 1b) reporting human extinction would be good, neither good nor bad, or bad.

Reasons why extinction would be bad or good/neutral

Human extinction would be **bad**…
(1 - The very weakest reason to 7 - The very strongest reason)

Table S2. Endorsements for reasons why human extinction would be bad.

Reason	Mean	SD
because my life would no longer be meaningful if humanity ended	2.92	2.01
because we would no longer have any chance of creating an ideal future society (utopia)	3.75	1.99
because it would prevent future people from having positive lives	4.80	1.86
because it would cause harm and pain to the humans that died from the extinction event	4.97	1.89
because we would have failed in our duty to past generations to preserve and advance the human species	4.98	1.83
because it would mean the loss of all human progress (cultural progress, technological progress, and other improvements to civilization)	5.45	1.62
because we would have failed in our duty to protect generations of humans that will exist in the future	5.45	1.61

Human extinction would be **good or neutral**…
(1 - The very weakest reason to 7 - The very strongest reason)

Table S3. Endorsements for reasons why human extinction would be good or neutral.

Reason	Mean	SD
because nothing matters anyway	2.43	1.77
because we are doomed anyway (there's nothing we can do to prevent human extinction)	2.98	1.81
because it would prevent further suffering for humans	4.04	1.98

because it would prevent humans causing further suffering for non-human animals	4.60	1.96
because it would prevent humans from further destroying the natural environment	4.90	1.98

Pushing extinction button

Imagine there was a button that only you could press. If you press the button, it would instantly end the lives of all humans painlessly (i.e., humanity would go extinct), though it would not end the lives of non-human animals, nor would it negatively affect the natural environment. Your decision is private - nobody else knows about the button.

Would you push the button?

- No: **91.9%**
- Yes: **8.1%**

Likeliest causes of extinction

If humanity went extinct in the next 100 years, which of the following would be the most likely causes?

Please rank from the most likely causes (at the top) to the least likely causes (at the bottom).

[ranked from 1 (most likely) to 7 (least likely)]

Table S4. Ranking the likelihood of different potential causes of human extinction in the next 100 years.[1]

Cause	Mean	SD
Nuclear war	2.22	1.30
Climate change	2.86	1.94
Global pandemic	3.18	1.39

[1] Notably, this data was collected in July 2022 during the height of the Russia/Ukraine war and before recent advances in artificial intelligence such as ChatGPT, which may have affected the rankings.

Meteor / asteroid	4.20	1.86
Insect decline	4.98	1.68
Volcano	5.25	1.37
Artificial Intelligence	5.30	1.77

Preventing extinction vs. suffering

If you had to choose, which would you prefer to do?

- Reduce the chance that humanity goes extinct in the next 100 years: **34.1%**
- Reduce the amount of suffering experienced by humans alive today: **65.9%**

Nihilism

Please read the following statement carefully and respond as truthfully and accurately as you can. There is no right or wrong answer.

"Despite our best efforts, nothing truly matters."

Strongly disagree (1) to Strongly agree (7)

Participants who previously responded that human extinction would be a **good** outcome:
$M = 4.50$ ($SD = 2.32$).

Participants who previously responded that human extinction would be a **bad** outcome:
$M = 2.36$ ($SD = 1.43$).

Independent t-test between the two: $t(12) = 3.14$, $p = .009$

Study 2: additional results

After participants completed measures about the badness and likelihood of human extinction and ranked the importance of several societal issues (which are all reported in the manuscript), they then responded to the following measures (with results following each measure):

Assumptions about human extinction

We are interested in how you were thinking human extinction would occur when answering the first questions of this survey (about the badness and likelihood of human extinction in the next 100 years).

Were you thinking that human extinction would involve a lot of suffering?

I was primarily thinking that …
(1) extinction would involve no suffering: **5.2%**
(2) neither (I had no clear assumption in mind): **31.1%**
(3) extinction would involve a great deal of suffering: **63.8%**

Were you thinking that human extinction would be slow/gradual or sudden/abrupt?

I was primarily thinking that …
(1) extinction would be slow/gradual: **37.4%**
(2) neither (I had no clear assumption in mind): **20.3%**
(3) extinction would be sudden/abrupt: **42.2%**

Were you thinking that extinction would occur through a catastrophic event (a sudden and extremely destructive event with far-reaching harmful consequences)?

I was primarily thinking that …
(1) extinction would NOT be a catastrophic event: **16.7%**
(2) neither (I had no clear assumption in mind): **17.9%**
(3) extinction would be a catastrophic event: **65.3%**

Were you thinking that extinction would happen because of events that killed all living humans, rather than some other way, like humans deciding not to have children?

I was primarily thinking that …

(1) extinction would happen some other way: **12.8%**
(2) neither (I had no clear assumption in mind): **9.6%**
(3) extinction would happen because of events that killed all living humans: **77.7%**

Were you thinking that the extinction event involved an event or process through which all humans (i.e., the species homo sapiens) will get replaced by a 'descendant species' (i.e., a new type of species or artificial entity) that would build on humanity's knowledge and culture?

I was primarily thinking that …
(1) extinction would NOT involve such a species replacement event/process: **64.5%**
(2) neither (I had no clear assumption in mind): **27.5%**
(3) extinction would involve such a species replacement event/process: **8.0%**

[only displayed if answered "3" on the prior question:]
Were you thinking the replacement process with the 'descendant species' was:
(1) voluntary (Humans agreed to be replaced): **0%**
(2) neither (I had no clear assumption in mind): **30.0%**
(3) involuntary (Humans didn't want to be replaced): **70.0%**

Causes of human extinction

We are interested in how you were thinking human extinction would occur when answering the first questions of this survey (about the badness and likelihood of human extinction in the next 100 years).

To what extent were you thinking about the following factors as a possible cause of human extinction?

(Note, we are not interested in whether you think these factors are plausible; we are only interested in whether you were **actively thinking about them** when answering the first questions of the survey.)

1 (I was not thinking about this factor at all) to 7 (I was strongly thinking about this factor)

Table S5. Ratings of the extent to which participants were actively thinking about various potential causes of human extinction when making judgments about its badness and likelihood in the next 100 years.

**Cause**	**Mean**	**SD**
Climate change / environmental degradation	5.26	2.04
War / conflict	4.88	2.26
Meteor / asteroid	3.99	2.29
Pandemic	3.90	2.08
Volcanic eruption	2.79	2.00
Artificial Intelligence	2.26	1.73
Infertility	1.83	1.35
Insect decline	1.61	1.28
Replacement with a descendant species	1.57	1.28

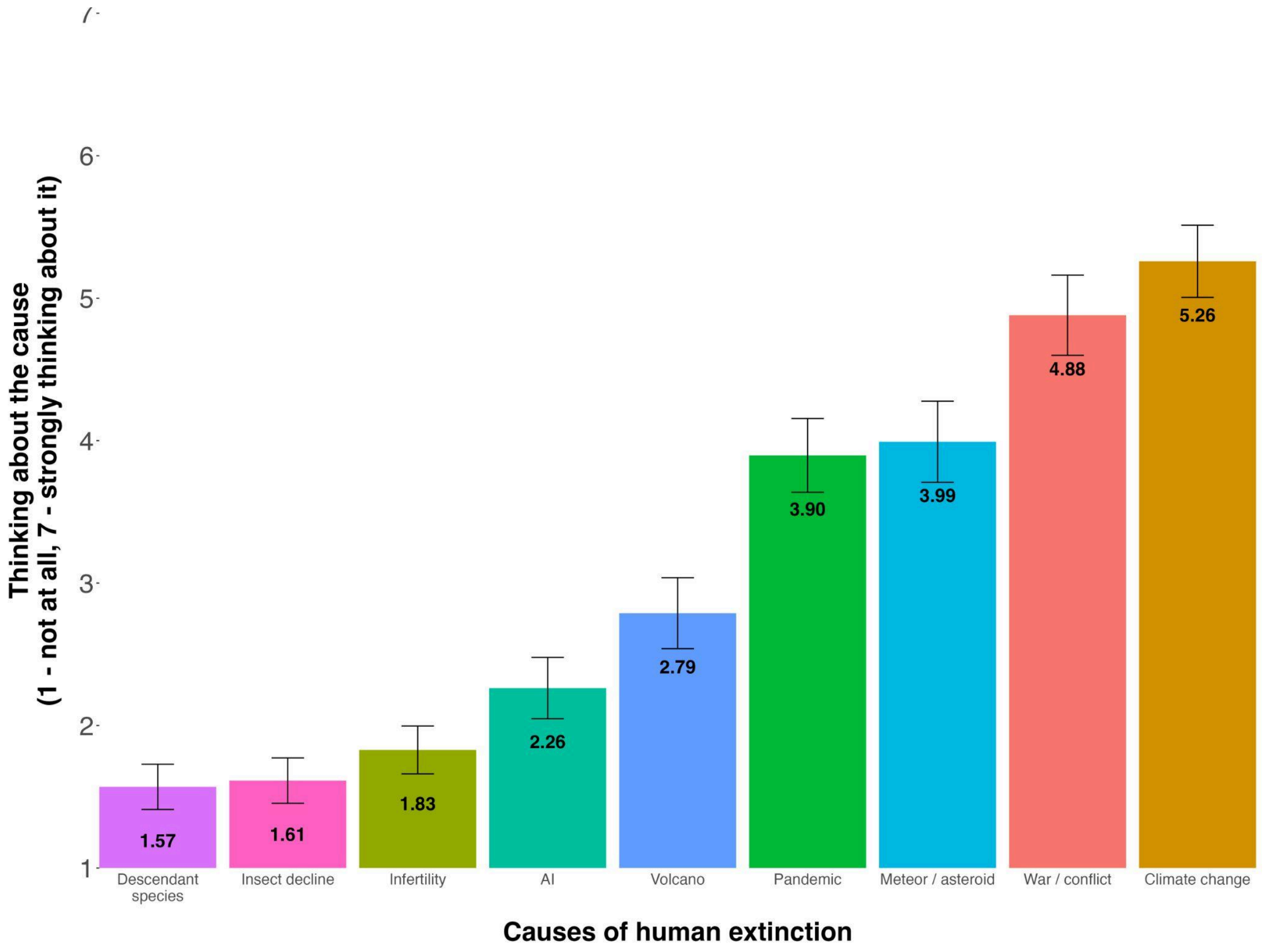

Figure S2. Ratings of the extent to which participants were actively thinking about various potential causes of human extinction when making judgments about its badness and likelihood in the next 100 years.

Badness of type of extinction events

For all scenarios, assume that

- the natural environment and non-human animals would remain unaffected.
- humanity is NOT being replaced by a 'descendant species'.

How bad would it be if the following type of extinction events were to happen in the next 100 years?

Not bad at all (1) - Extremely bad (7)

- Immediate **painful** death of all existing humans: ***M*** **= 5.92 (*SD* = 1.41)**

- Gradual **painful** death of all existing humans: ***M* = 6.31 (*SD* = 1.21)**
- Immediate **painless** death of all existing humans: ***M* = 4.17 (*SD* = 2.10)**
- Gradual **painless** death of all existing humans: ***M* = 4.75 (*SD* = 1.85)**

Tractability for different actors

To what extent do you think the following types of actors could, if they wanted, take actions to meaningfully reduce the risks of human extinction?

1 (Not at all) to 7 (To a large extent)

Table S6. Ratings of the tractability of certain types of actors to reduce the risks of human extinction.

**Actors**	**Mean**	**SD**
Governments	5.78	1.51
For-profit companies	4.87	1.91
Social movements	3.99	1.69
Non-profit organizations	3.90	1.56
Influential individuals	3.89	1.75
Individual typical citizens	3.04	1.75

Study 3: additional results

Table S*7*. Descriptives and t-test against the midpoint for each of the current societal issues used in the relative prioritization of human extinction.

**Societal issue**	**Condition**	**Mean (SD)**	**One-sample t-test against midpoint**
Healthcare	Control	0.19 (1.81)	$t(509) = 2.33$, $p = .020$
Healthcare	Intervention	0.22 (1.84)	$t(489) = 2.70$, $p = .007$

Education	Control	0.36 (1.88)	$t(509) = 4.33, p < .001$
Education	Intervention	0.53 (1.85)	$t(489) = 6.41, p < .0001$
Law and order	Control	0.51 (1.64)	$t(509) = 7.06, p < .001$
Law and order	Intervention	0.70 (1.72)	$t(489) = 8.96, p < .0001$
Transportation	Control	1.10 (1.72)	$t(509) = 14.43, p < .0001$
Transportation	Intervention	1.12 (1.71)	$t(489) = 14.56, p < .0001$
Homelessness	Control	0.53 (1.81)	$t(509) = 6.55, p < .0001$
Homelessness	Intervention	0.63 (1.82)	$t(489) = 7.63, p < .0001$
Aggregate of all five	Control	0.54 (1.44)	$t(509) = 8.43, p < .0001$
Aggregate of all five	Intervention	0.64 (1.49)	$t(489) = 9.53, p < .0001$

Additional figures

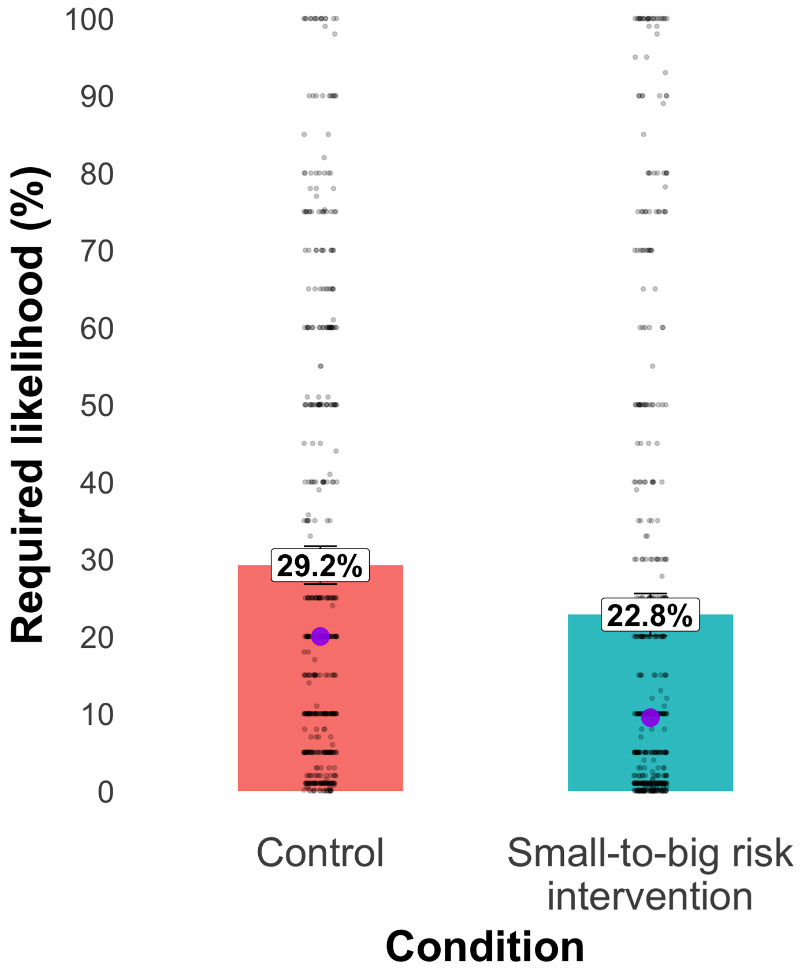

Figure S3. Required likelihood for the risk of human extinction this century to be the top societal priority, by control versus small-to-big risk intervention (Study 3). Error bars indicate 95% CIs.

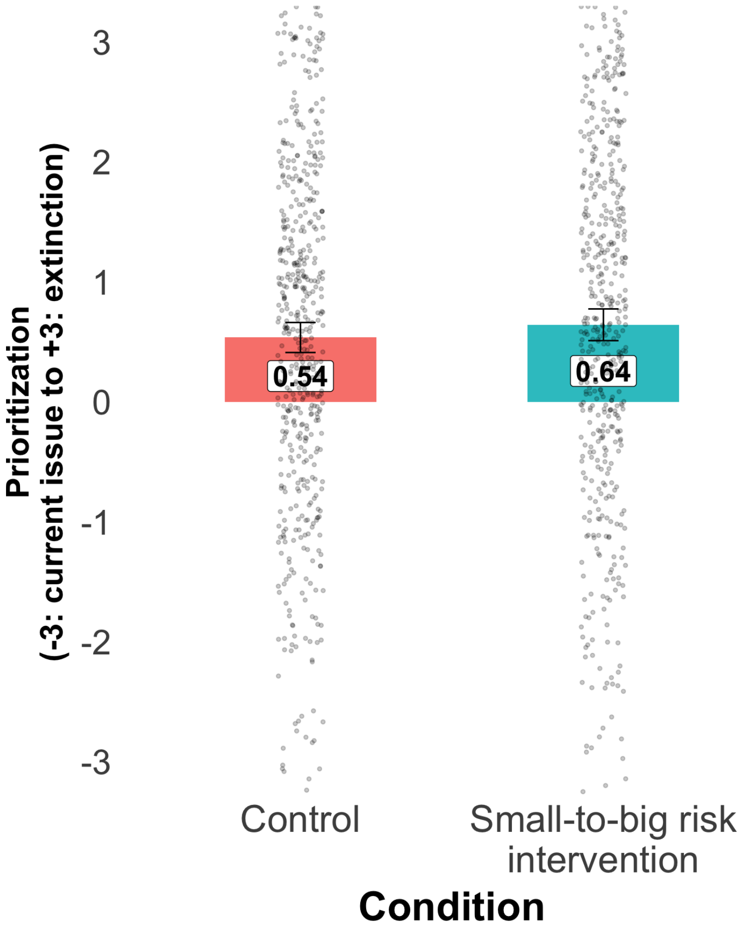

Figure S4. Prioritization of human extinction versus current societal issues, by control versus small-to-big risk intervention (Study 3). Error bars indicate 95% CIs.

Study 4: additional results

Correlation between likelihood estimate of human extinction this century and tractability of reducing risks of human extinction: $r = .01$, $p = .85$ (control condition only).

Table S8. Descriptives and t-test against the midpoint for each of the current societal issues used in the relative prioritization of human extinction.

Societal issue	Condition	Mean (SD)	One-sample t-test against midpoint
Healthcare	Control	-1.11 (1.68)	$t(298) = -11.50$, $p < .0001$
Healthcare	Intervention	-1.14 (1.65)	$t(268) = -11.37$, $p < .0001$
Education	Control	-0.92 (1.77)	$t(298) = -9.00$, $p < .0001$
Education	Intervention	-1.04 (1.77)	$t(268) = -9.62$, $p < .0001$
Law and order	Control	-0.37 (1.77)	$t(298) = -3.60$, $p < .001$
Law and order	Intervention	-0.35 (1.68)	$t(268) = -3.45$, $p < .001$
Transportation	Control	-0.19 (1.74)	$t(298) = -1.86$, $p = .064$
Transportation	Intervention	-0.08 (1.79)	$t(268) = -0.75$, $p = .455$
Homelessness	Control	-0.80 (1.71)	$t(298) = -8.09$, $p < .0001$
Homelessness	Intervention	-0.81 (1.75)	$t(268) = -7.55$, $p < .0001$
Aggregate of all five	Control	-0.68 (1.42)	$t(298) = -8.24$, $p < .0001$
Aggregate of all five	Intervention	-0.69 (1.39)	$t(268) = -8.10$, $p < .0001$

Additional figures

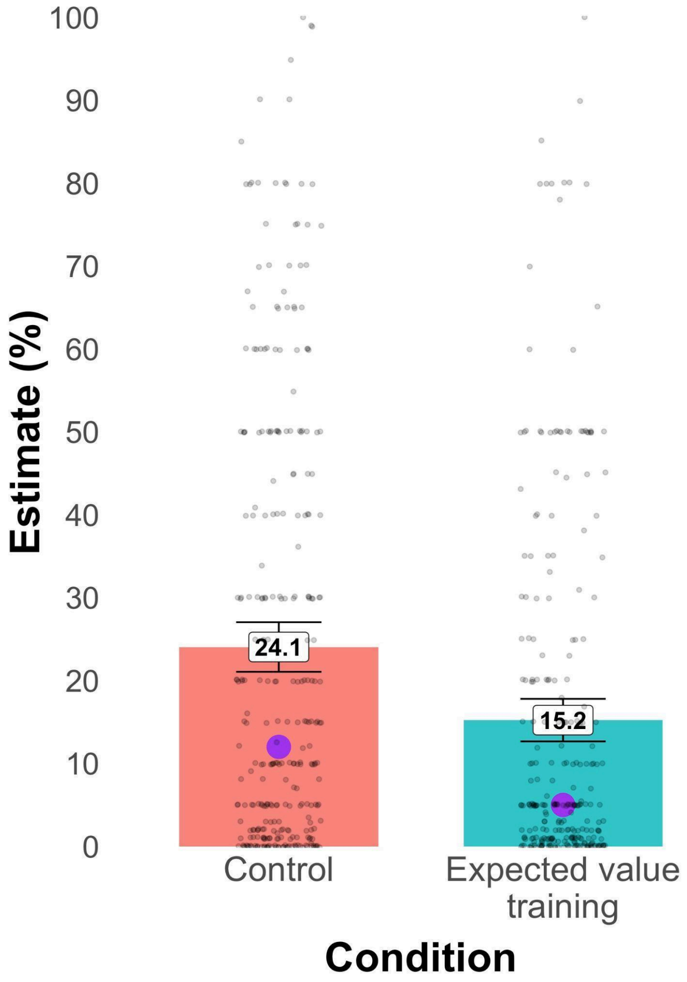

Figure S5. Likelihood estimates of human extinction this century, by control versus expected value training (Study 4). Error bars indicate 95% CIs.

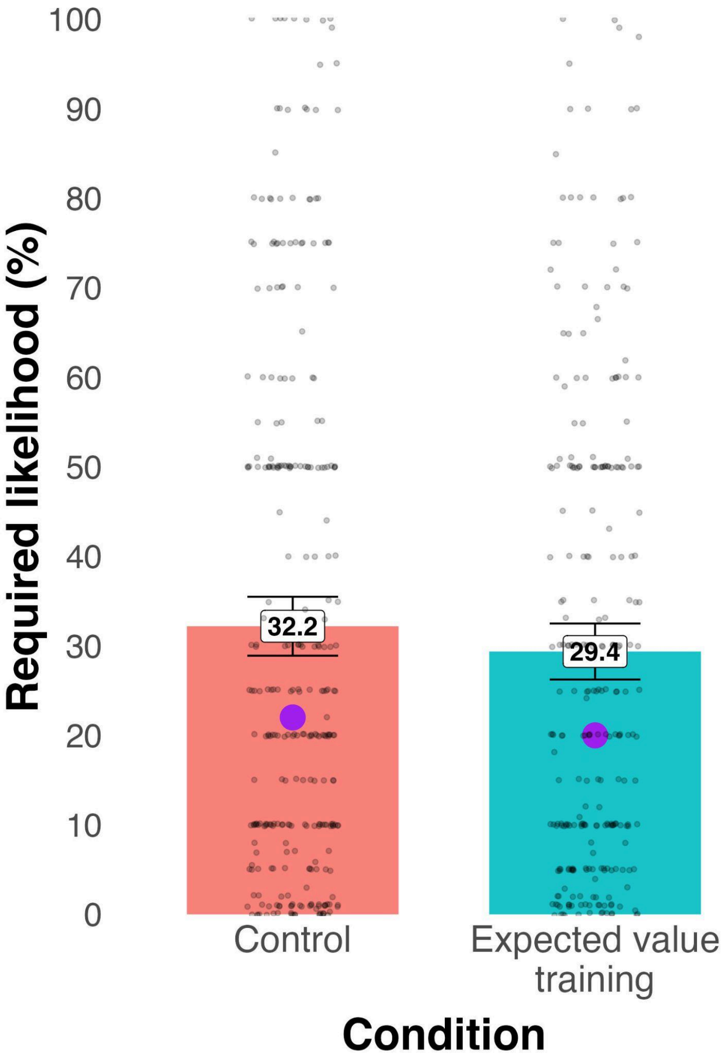

Figure S6. Required likelihood for the risk of human extinction this century to be the top societal priority, by control versus expected value training (Study 4). Error bars indicate 95% CIs.

Study S1

Methods

Participants were recruited through Amazon Mechanical Turk (mTurk) as part of a larger study approved by the University of Oxford Central University Research Ethics Committee. A total of 303 U.S. participants were recruited. Fifty-three participants were excluded for failing the attention check and two participants were excluded for not accepting the study assumptions, leaving a final sample of 248 participants (105 female, M_{age} = 38.87 years, SD_{age} = 10.56). Participants were asked “How bad would you find it if humanity (in our world) went extinct relatively soon?” and responded on a 7-point Likert scale from *1 (not bad at all)* to *7 (extremely bad)*.

Results

The average response was significantly above the scale midpoint (M = 5.41, SD = 1.89; $t(247) = 11.74$, $p < .0001$, d = 0.75, 95% CI = [5.17, 5.64]). 70.5% of participants responded above the midpoint. More specifically, 6.9% responded with “1”, 3.6% responded with “2”, 6.5% responded with “3”, 12.5% responded with “4”, 9.3% responded with “5”, 18.1% responded with “6”, and 43.1% responded with “7”. These results demonstrate that most people view human extinction as a bad outcome, although it is noteworthy that people differ in *how* bad they think it would be.

Study S2

Methods

Participants were recruited through mTurk as part of a larger study approved by the Northeastern University Institutional Review Board. A total of 397 U.S. participants were recruited (148 females, M_{age} = 40.70 years, SD_{age} = 12.37). Participants were asked to rank a list of six societal issues in terms of their importance for society to prioritize. The issues were presented in a randomized order and included human extinction, healthcare, poverty, education, law and order, and climate change. Participants were instructed to rank the issues from *1 (most important)* to *6 (least important)*.

Results

Participants ranked healthcare as the most important (M = 2.67, SD = 1.33), followed by poverty (M = 3.17, SD = 1.50), climate change[2] (M = 3.36, SD = 1.80), education (M = 3.54, SD = 1.39), law and order (M = 4.01, SD = 1.73), while human extinction was ranked as the least important (M = 4.25, SD = 1.93)[3].

---

[2] It is possible that many participants considered climate change a human extinction risk. Thus, once accounting for climate change, it is possible that participants prioritize human extinction more than the results suggest.

[3] Notably, there was a bimodal distribution such that 18.4% ranked it as their most important priority and 40.3% ranked it as their least important priority. No other societal issue was as polarizing. We speculate this result is because some participants' reasoning approximated expected value, but most participants' reasoning did not.
